# Development of a relationship counselling website to identify and mitigate risk of intimate partner violence in the context of women’s PrEP use

**DOI:** 10.1371/journal.pdig.0000329

**Published:** 2023-08-14

**Authors:** Miriam Hartmann, Sarah T. Roberts, Noah Triplett, Siyanda Tenza, Onthatile Maboa, Lydia Mampuru, Nonkululeko Mayisela, Dorica Mbewe, Elizabeth E. Tolley, Krishnaveni Reddy, Thesla Palanee-Phillips, Elizabeth T. Montgomery

**Affiliations:** 1 Women’s Global Health Imperative, RTI International, Berkeley, California, United States of America; 2 Department of Global Public Health, Karolinska Institutet, Stockholm, Sweden; 3 Wits Reproductive Health and HIV Institute, Faculty of Health Sciences, University of the Witwatersrand, Johannesburg, South Africa; 4 FHI360, Durham, North Carolina, United States of America; 5 University of Washington, Department of Epidemiology; School of Public Health, Seattle, United States of America; Iran University of Medical Sciences, IRAN (ISLAMIC REPUBLIC OF)

## Abstract

Discreet, accessible interventions are urgently needed to mitigate the risk of intimate-partner violence (IPV) and other relationship barriers that women encounter to using HIV prevention methods such as pre-exposure prophylaxis (PrEP). We adapted a counsellor-administered intervention, CHARISMA, into a mobile-optimized website to enhance accessibility and reduce human resources required for HIV prevention and relationship counseling. Using human-centered design and participatory methods, CHARISMA was adapted through workshops with former CHARISMA in-person intervention participants (n = 14; ages 18–45) and web development ‘sprints’ combined with cognitive interviews (n = 24). ‘CHARISMA mobile’ was then beta-tested with 81 women naïve to the in-person intervention. In beta-testing, participants used a ‘think aloud’ process to provide feedback on ease of use and rated design, functionality, comprehension, confidentiality, safety, and usefulness on a scale of 1 to 5 via a survey. Data were conducted in four rounds, interspersed with rapid assessment according to go/no-go criteria, and website adaptations. The updated website was pilot tested for ‘real-world’ feasibility and acceptability among 159 women using their own smartphones at a location of their choice. Feedback was measured via surveys and website analytics. Workshops and cognitive interviews generated insights on technology use, contextual adaptations, and confidentiality, which were integrated into the beta version. The beta version met all ‘go’ criteria and was further adapted for pilot testing. In pilot testing, users found the website was useful (mean rating 4.54 out of 5), safe (4.5 out of 5), and had few concerns about confidentiality (1.75, representing low concern). On average, users rated the website more than 4 stars out of 5. Beta and pilot-testing suggested the smartphone-optimized website was well-accepted, relevant, engaging, feasible to administer, discreet and safe. Results contributed to a refined website, suitable for adaptations to other contexts and further evaluation where outcomes related to PrEP use and relationships should be assessed.

## Author summary

Relationship-based issues such as intimate partner violence remain a barrier for women to effectively use HIV prevention like pre-exposure prophylaxis (PrEP), and limited tools or human resources exist to provide adequate counselling on this topic. We involved women in the adaptation of a novel in-person counselling program addressing relationship-based barriers to PrEP to a mobile website. Using a multi-step process we co-designed the website with women, beta and pilot tested it to identify barriers and facilitators to use and improve its features and content. The process helped us design a mobile website that was contextually relevant, feasible, and easy to use. The website should be further tested for its impact on women’s lives as it relates to their PrEP use within relationships.

## Introduction

Oral pre-exposure prophylaxis (PrEP) is an effective method approved for use as an HIV prevention option [1] that has been rapidly expanding in availability across sub-Saharan Africa. While most sub-Saharan African countries, including South Africa, have included oral PrEP in their HIV prevention programs, uptake and use is lower than ideal to tangibly reduce HIV transmission rates [2,3]. Evidence indicates that uptake and use of PrEP among women is influenced by a range of social and relational factors, including relationship dynamics with male partners and challenges related to disclosure of use of HIV prevention methods. Inequitable gender norms around decision-making and fear or experience of partner-related violence limit women’s PrEP use. This challenge to PrEP use is particularly salient in contexts where PrEP is seen as potentially threatening to relationships, because it might be perceived to indicate mistrust or infidelity in a relationship or be misinterpreted to mean the user is actually HIV positive given PrEP is antiretroviral drug-based [4–10].

Multiple interventions or strategies have been developed to mitigate relationship-based barriers to women’s PrEP use. They include individual counselling and support groups for young women using PrEP to address gender norms, partner disclosure, and intimate partner violence (IPV), as well as community sensitization efforts to address harmful norms and attitudes about PrEP use among men and the broader community. [11–17] However, these approaches take significant time and require sustained use of human resources to implement, especially in the public sector. They also face structural challenges to implementation. In particular, healthcare providers and other key influencers in communities often have a poor understanding of IPV, may accept/normalize IPV, and are hesitant to address complex or sensitive time-consuming issues. Public health clinics also often lack private and confidential spaces, which are critical when responding to effective management of victims of violence [18].

Given constraints of the public health care sector, and evidence that mobile or digital tools can offer IPV related support to women discretely [19–21], offering relationship-based PrEP support to women via a mobile health (mHealth) tool may be an appropriate solution. In South Africa, mobile phone/online coverage is as high as 90% across the country, so mHealth tools are likely to be accessible to most women who could benefit from their use [22,23]. In this study, we aimed to adapt CHARISMA, an in-person empowerment-based counselling intervention [12,13], for self-administered delivery through a mobile device optimized website, and evaluate its feasibility and acceptability of delivery. CHARISMA was chosen as it was the only individually delivered intervention addressing relationship-based barriers to PrEP use available at the time and already included some electronically delivered components, such as a relationship-assessment tool and videos elucidating key counselling topics, that had been found to have high acceptability. The intervention’s grounding in an empowerment model, which has been effective in other IPV interventions [24], was also deemed a strength given the theoretical linkage between a lack of empowerment via gender inequity and limitations to women’s decision-making power in sexual and reproductive health domains and beyond [25,26]. We had an added goal of making it available through a National Department of Health (NDoH) sponsored website in South Africa that may be paired with future rollout efforts for all PrEP methods (e.g. the dapivirine vaginal ring, injectable cabotegravir) [27] and other medical delivery services impacted by gender-based violence (e.g. HIV treatment adherence [28], contraceptive use [29]).

## Methods

### Overall study design and participants

**Fig 1:**
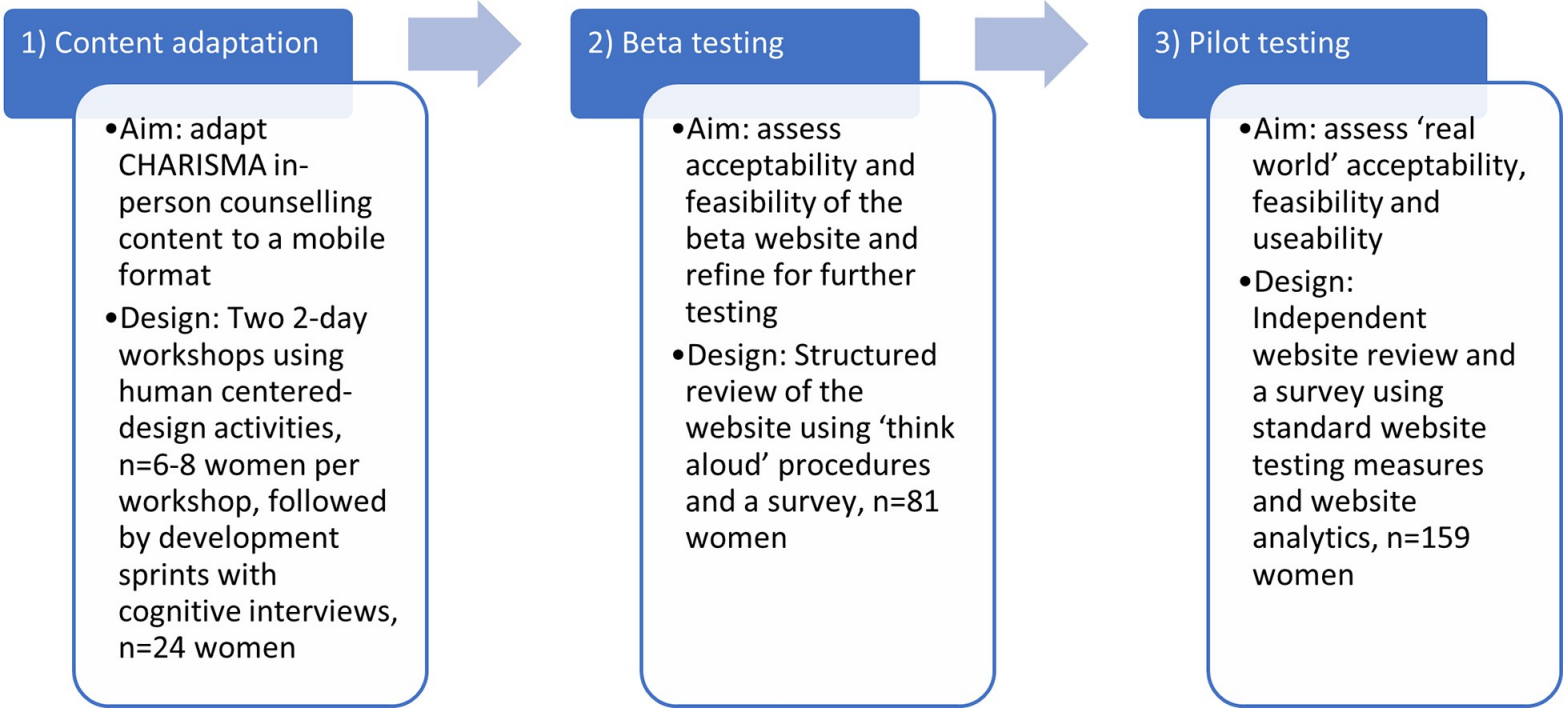
Overview of CHARISMA mobile website development process.

The development and testing of the CHARISMA mobile website included three distinct phases of work: 1) adaptation and translation of the in-person counselling content into mobile content, 2) beta testing (i.e. feedback on the website while in production), and 3) pilot testing. Fig 1 illustrates these phases, noting key methods and sample sizes. All phases were conducted in Johannesburg, South Africa and included women ages 18–45 –disaggregated by age group (adolescent girls and young women (AGYW), 18–24, and older women, 25–45)–who were either using or interested in using PrEP. The work was reviewed by the University of the Witwatersrand Human Research Ethics Committee (HREC) and assigned a non-research determination. All participants provided oral consent prior to engaging in website development assessment or evaluation activities. Further details of the methods used for each phase are described below.

#### Phase 1. Content adaptation and mobile site development

Content adaptation and development included participatory workshops and ‘development sprints’ (i.e. individual website development milestones to be completed and tested in short timeframes) interspersed with cognitive interviews.

Two day workshops (n = 2) were conducted separately with AGYW (n = 6) and adult women (n = 8), all of whom had prior experience with the CHARISMA in-person intervention [12], in November and December 2020. Participants were selected to represent those who had experience with each of the three counselling topics covered by CHARISMA, i.e., partner communication, PrEP disclosure, and IPV, using study records and staff knowledge of the participants. The agenda primarily included participatory activities drawing from human-centered design (HCD) such as persona creation, prototyping, and user journey mapping [30]. Within these workshops women were asked to discuss their current mobile technology use, including barriers to use, the CHARISMA content and how it could be adapted to a mobile website, as well as confidentiality and safety concerns related to using the intervention’s relationship-assessment quiz and targeted counselling materials on topics such as IPV, which could result in social harms if seen by others. The workshops were co-facilitated by the CHARISMA research team and collaborating partners with experience developing and running several South African NDoH mobile platforms related to PrEP. Members of the technical website development team were also present at the workshops to obtain women’s feedback and ideas in real time to inform the website development.

Following completion of the workshops, the website development team held an inception meeting with the full CHARISMA research team in February 2021 where workshop feedback was reviewed, along with key goals and metrics for website development and evaluation. A schedule of ‘sprints’ was established. During the completion of these sprints, 24 women naïve to the CHARISMA intervention who were recruited from those involved in other non-HIV related research at the clinic site, were invited to participate in cognitive interviews to review pieces of content. These interviews sought women’s feedback on the look, feel and aesthetic appeal of the website, the language used, and on the use of key safety features such as log-ins for secured access and to protect confidentiality of user input. Feedback was incorporated into the website leading to an English-only beta version of the site.

#### Phase 2. Beta testing

The CHARISMA mobile content was beta tested from July to August 2021 among 81 women, including 55 AGYW ages 18–24 and 26 women ages 25–45. Eligible women were naïve to the CHARISMA intervention, sexually active, either eligible for PrEP or current PrEP users, and spoke English. Potential participants were identified by Wits Reproductive Health and HIV Institute (Wits RHI) outreach staff, primarily through contacting women who participated in a cross-sectional sexually transmitted infection study at Wits RHI.

Facilitators led participants in one-on-one beta testing sessions using observational feedback interviews in which women completed the entire web-based intervention using a ‘think aloud’ process on a study-owned tablet. The facilitator walked the participant through the mobile beta version step by step, asking for feedback on the usability and functionality of the intervention, including what the participant liked and did not like about each feature. Similar to a cognitive interview process, participants were asked to describe their thoughts, feelings, and ideas about the mobile content as they moved through the intervention starting with administration of a relationship quiz (called the HEAlthy Relationship Tool or “HEART” [31,32]) followed by the counseling module content that was recommended based on their relationship circumstance. In addition, close-ended questions assessed the design and functionality of the mobile site (e.g., performance, ease of use, navigation, gestural design, graphic design) as well as opinions about the site style and format, comprehension of the content, concerns around confidentiality and safety, and the potential usefulness of the intervention. These questions were drawn from the Mobile App Rating Scale (MARS) [33] and other similar resources such as the ‘net promoter score’ [34]. We also assessed overall satisfaction and potential usefulness of the content. Interviews were conducted in a series of 4 rounds of 15–24 interviews each, after which results were descriptively analyzed and assessed according to go/no-go criteria (specified in Table 2 in the results section), and problems addressed through website adaptations iteratively throughout the process. The first column of Table 1 presents key dimensions measured in the beta testing phase, along with illustrative questions.

**Table 1:** Key constructs and evaluation measures from beta and pilot testing phases.

Construct	Evaluation measures	
	Beta test	Pilot test
**Engagement / Usability**	Whether the site is perceived to be interesting, engaging, entertaining for oneself and others. *‘Is the website interesting to use*? *Does it use any strategies to make it engaging by presenting its content in an interesting way*?*’* [33]	Amount of time each participant spent on mobile website, itemization of CHARISMA content components accessed, and time spent engaging with each–these will be assessed through mobile metrics (e.g. Google Analytics) and through participant self-report.
**Functionality / Feasibility**	Problems with links, speed or navigation noted by participants or observed. *‘How was the process of learning to navigate the site*? *How accurate or fast was the site*?’ [33]	Challenges encountered with accessing mobile content at home or in other locations; any mobile functionality challenges
**Aesthetics**	Desired look for the site, including graphics, buttons/icons/content. *‘Is arrangement and size of buttons/icons/menus/content on the screen appropriate or zoomable if needed*?*’* [33]	Attractiveness of the website. *‘The website has a clean and simple presentation’* [35]
**Information**	Perception of the information on the site and how it is displayed. *‘Is visual explanation of concepts–through charts/graphs/images/videos*, *etc*.*–clear*, *logical*, *correct*? ‘ [33]	Clarity of written and visual content. *‘It was clear to me what this website can do for me*.*’* [36]
**Safety** *	Assessment of women’s concerns about site safety, e.g., feeling ‘safe’ or ‘very safe’ using the content Rating of items such as, *‘I would feel safe using the website’*	
**Confidentiality** *	Assessment of women’s concerns about data confidentiality Rating of items such, *‘I would be worried that other people could see the information I enter into the website’*	
**Overall satisfaction / Acceptability** *	Women’s overall satisfaction with the site, e.g. overall rating, would they use it in the future, and would they recommend it to others. *‘What is your overall star rating of the website*?*’* [33] *‘How likely are you to recommend the website to a friend or colleague*?*’* [34,35]	

* The same safety, confidentiality, and overall satisfaction metrics were used across Beta and Pilot testing phases.

#### Phase 3. Pilot testing

Pilot testing of a revised mobile website took place from November to December 2021 with 159 women, including AGYW ages 18–24 (n = 73) and adult women ages 25–45 (n = 86). Women were recruited at public health clinics, through social media, and using peer-to-peer recruitment and met the same eligibility criteria as those involved in beta testing. The emphasis of this phase was to assess ‘real world’ acceptability and feasibility and as such, participants were asked to access the website on their own device at a location of their choice, take the HEART quiz, and browse the content that was interesting to them. If they chose to access the website at the Wits RHI clinic, which offered free-WiFi, participants were provided a private and quiet space where they could review the website on their own. Following website review, all participants completed a brief interviewer-administered survey either face-to-face at the clinic or over the phone. The survey included sociodemographic information, items on where and how the participant accessed the website, their general access to cellphone data, and their experience using the website. The latter questions drew on the Single Ease Question (SEQ) [37] to measure ease of use, the Standardized User Experience Percentile Rank Questionnaire (SUPR-Q) [35], which measures useability, credibility, appearance, and loyalty, ‘usefulness’ sub-items from the Adoption Likelihood Factors Questionnaire (AFLQ), and the Net Promoter score, which measures anticipated promotion of the website to others [36]. Finally, a CHARISMA-specific composite score measured how much the website was perceived as meeting needs around relationship issues and PrEP use. Table 1 presents the pilot testing phase constructs with illustrative questions. Website analytics were used to assess time spent on the website and pages visited. Data was analyzed using SPSS.

Participants in all phases of research (workshops, cognitive interviews, beta and pilot phases) were reimbursed R50-100 for their time and expense travelling to and from the clinic. Additionally, lunch was provided for the participants during the workshops.

## Results

The results of the three phases of mobile website development and testing are described below.

### Phase 1. Content adaptation

Feedback from the workshops and cognitive interviews fed into three main decision areas for the website development: 1) technology decisions, 2) content adaptations/desires, and 3) confidentiality and safety considerations.

Firstly, in terms of technology, participants described having smartphones and using various social media platforms such as TikTok, Instagram, WhatsApp, and Facebook. Platforms used were similar across both age groups (i.e. 18–24 and 25–45), however TikTok was unique to younger women. Both groups described having limited storage space on their phones and one group explained how WhatsApp, which was described as “the oxygen of the phone,” (i.e. essential for connection in life) had to be deleted to download Zoom for the project’s pre-workshop call and then re-downloaded once Zoom could be deleted. This led to a decision to develop a mobile website rather than an app, which therefore wouldn’t need to be downloaded.

**Fig 2:**
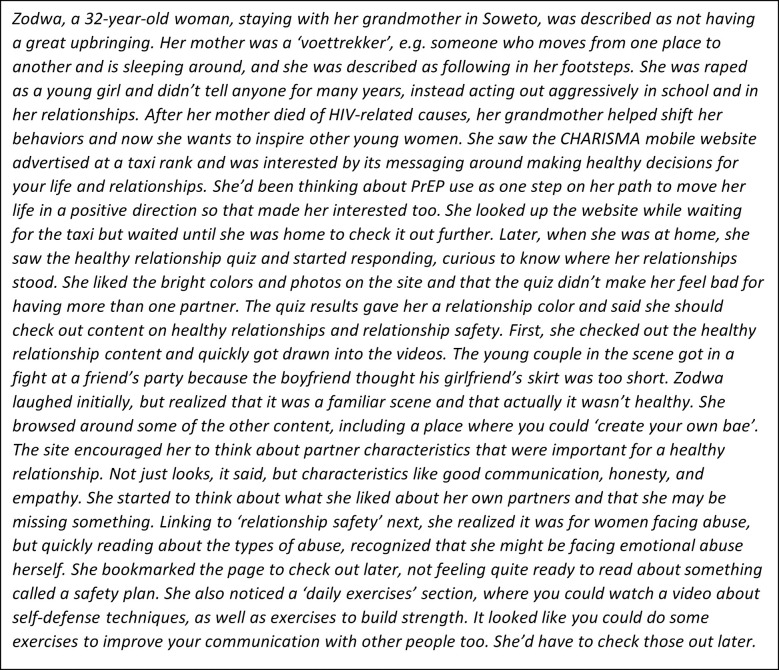
Illustrative participant-developed website journey for persona, ‘Zodwa’.

Progressive activities–developing personas of website users, creatively re-designing each of the CHARISMA counselling topics, and the development of a journey map describing the experience of one persona using the site, from initial awareness through her use of all features–contributed to content development. The description of Zodwa’s journey with the website (Fig 2), created by workshop participants, highlights several key inputs. These included framing the site towards women’s desires for healthy relationships, a need for interactive content, and words and phrases that may resonate with young, urban South Africans.

Women also discussed confidentiality and safety of the site with most women expressing the opinion that they would feel safe using their mobile phones to access website content. To further enhance a sense of safety, they suggested having terms and conditions, which would describe confidentiality measures. They felt that logins should be automatically logged out after a period of time and that any request to receive your relationship quiz results should be sent as a password protected pdf.

**Fig 3:**
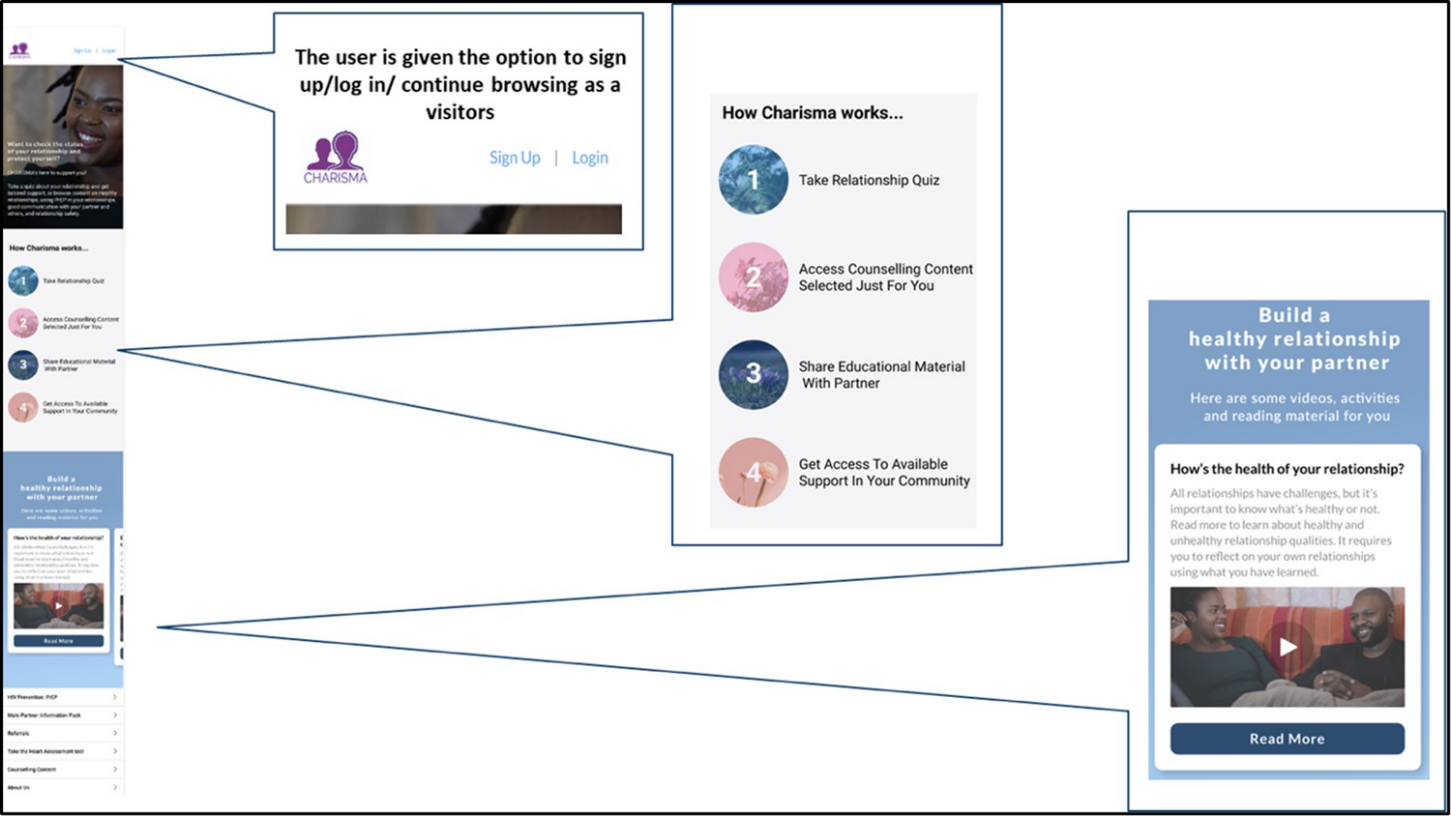
CHARISMA-mobile beta website homepage.

Fig 3 illustrates some scenes and features of the beta website refinements based on women’s input, for example the home screen, a visual of how the website will work, and an image of one counselling content area (i.e. healthy relationship content). Other suggested interactive content such as daily mood check-ins, the ability to make direct calls/messages and to geolocate service providers, and the ability to share information on social media was not integrated due to resource constraints.

### Phase 2. Beta testing

Across all measured domains, beta testing showed that the mobile phone-optimized site was generally well-accepted, relevant, engaging, and feasible to administer. These findings were consistent across both age groups (18–24 & 25–45) as seen in Table 2, and across all counselling topics received. In qualitative discussions and observations, some functionality issues were identified, though these did not strongly impact feedback on the site. Most functionality issues, such as videos not playing and slow page loading, were addressed in between beta testing rounds. Participants did not raise serious safety or confidentiality concerns either qualitatively or quantitatively. In the qualitative discussions, most participants described that no one would see them using the website, that their phones were their private property, or that they had passwords on their phones.

**Table 2:** Beta-testing results by category of the go/no-go criteria.

Domain	Preferred Target	Mean Scores (Standard Deviation) or Percentage			Result
		AGYW	Adult women	Total	
**HEART self-administration**	HEART can be comprehended and self-administered in < = 10 minutes by >60% of AGYW and women	83% comprehended without repetition; 56% self-administered in < = 15 minutes	81% comprehended without repetition; 35% self-administered in < = 15 minutes	83% comprehended without repetition; 49% self-administered in < = 15 minutes	Met minimum acceptable target (i.e. comprehension and self- in < = 15 minutes)
**Video acceptability**	Average score of ≥4 for videos relevance and entertainment	4.27 (0.575)	4.54 (0.314)	4.37 (0.510)	Met preferred target
**Counseling content acceptability**	Average score for site content relevance and entertainment ≥4	4.48 (0.483)	4.42 (0.572)	4.46 (0.510)	Met preferred target
**Functionality**	Average score of ≥4 for survey measures with no problems with links, speed or navigation observed by staff or participants	4.40 (0.693)	4.57 (0.477)	4.46 (0.632)	Met preferred target
**Aesthetics**	Average score of ≥4 for CHARISMA content and graphics	4.16 (0.697)	4.37 (0.576)	4.23 (0.664)	Met preferred target
**Counseling content information**	Average score of ≥4 for interpretation of counselling information	4.46 (0.553)	4.73 (0.323)	4.55 (0.505)	Met preferred target
**Safety**	Majority of participants ‘strongly agree’ or ‘agree’ that they feel “safe” using site, indicated by average score of ≥4	4.56 (0.604)	4.58 (0.857)	4.56 (0.691)	Met preferred target
**Confidentiality**	No participants raise concerns about user confidentiality, indicated by average score of ≤2	1.75 (0.758)	1.85 (1.06)	1.78 (0.860)	Met preferred target
**Overall quality**	Users would recommend the site to many people or everyone, would use the site in the future, and rate it 4 or 5 stars	4.22 (0.535)	4.49 (0.454)	4.30 (0.523)	Met preferred target

Other areas of feedback related broadly to the themes of content accessibility and desire for individualization and further visualization of content. For instance, several participants had trouble finding the healthy relationships counselling content as it was the one topic area that the HEART relationship quiz results did not link to directly, and some noted that it was hard to read through long paragraphs and that the amount of information was overwhelming. However, others found it well arranged without too much information per paragraph or being too complicated. The one website section that participants found challenging was the tailored counseling recommendations page, which summarized the HEART relationship quiz scores, interpreted the results for participants, and recommended one of three tailored counseling modules (e.g., partner communication, PrEP disclosure, and IPV). A substantial portion of participants found the results hard to interpret, and requested a more personalized presentation of results (referring to the participant directly, in the second person, instead of “other women” who have similar scores) and a clearer visual representation of the scores. Some participants also found the section on male partner concerns and responses to be difficult to interpret and wanted a clearer link between the concerns and the potential responses (e.g., through itemized bullets or a table). Based on this feedback, the largest change made to the site was the integration of a visual representation of the HEART scores using color coded dots. Other changes included linking the healthy relationship content at the bottom of all other counselling topics, revising the formatting of the ‘common male partner concerns about PrEP’ table, and changing various word choices to enhance understandability (e.g., finances to money, frightened to scared, digitize to ‘put online’).

### Phase 3. Pilot testing

All pilot testing participants reported being Black cis-gender women. The vast majority had completed secondary school with a greater proportion of the older women having completed tertiary education (36% vs. 25%). Almost all participants reported being heterosexual (96%), most had one partner currently (82%), and the majority had never used PrEP, but were interested in doing so (91%).

Participants largely accessed the CHARISMA site either at their own or someone else’s home (48%) or at the Wits RHI clinic (39%). A smaller proportion accessed the site at a public or other location, such as school or a library (13%). Approximately 70% of participants accessed the site using wifi, with only 30% accessing using a cellphone data plan. Those using a cellphone data plan, reported that they were largely prepaid. Most participants said they can afford cellphone data when they need it (66%) and most said their reception at home was good (77%).

Based on website analytics from the study period, there were 200 visitors from South Africa, which is greater than the pilot study sample. Among these visitors, the average time users engaged with the site was 14 minutes 36 seconds and the average number of engaged sessions was 1.5, indicating repeat visits. Almost three quarters of visitors (73%)–visited the HEART relationship quiz with 88% of these reaching the results page. One fifth of users (20%; 40 users) visited the healthy relationship page (77 times), 22% (44 users) visited the partner communication page (75 times), 28% (56 users) visited the PrEP disclosure page (101 times), and 14% (27 users) visited the intimate partner violence page (51 times). About a quarter (22%; 45 users) accessed the referrals page (121 times), 27% (53 users) accessed a page on resources for your male partner (114 times), and 17% (33 users) accessed additional information on PrEP (65 times).

Questionnaire responses about the website indicated high acceptability of the site (Table 3). The average Single Ease Question (SEQ) was 6.18 out of 7. The SUPR-Q, indicated high scores across all domains with an average overall score of 4.61 out of 5. Average usefulness (4.53 out of 5), safety (4.5 out of 5), and confidentiality (1.75 indicating few concerns) scores were similarly positive. The average CHARISMA-specific composite score was 4.43 out of 5. On average participants reported they would share the website details with many people or everyone per the net promoter score and that they would rate that site 4.6 out of 5 stars. CHARISMA-specific, safety and confidentiality scores were similar to those seen in beta testing. Results were similar for both age categories.

**Table 3:** Pilot testing website assessment results.

	18–24 years	25+ years	Overall
	N	N	N
SEQ score, mean (SD)	6.21 (1.31)	6.15 (1.29)	6.18 (1.30)
Raw SUPR-Q (SD)	4.63 (0.45)	4.59 (0.56)	4.61 (0.51)
Net promoter score^+^	4.52 (0.75)	4.44 (0.90)	4.48 (0.83)
‘Usefulness’ score from AFLQ, mean (SD)	4.54 (0.39)	4.53 (0.38)	4.53 (0.39)
Safety score, mean (SD)	4.51 (0.53)	4.49 (0.55)	4.5 (0.54)
Mean confidentiality score (SD)	1.75 (0.77)	1.75 (0.84)	1.75 (0.81)
Mean CHARISMA specific composite score (SD)	4.42 (0.51)	4.46 (0.51)	4.43 (0.51)
Overall star rating	4.47 (0.76)	4.7 (0.58)	4.6 (0.68)

Legend: Note that all scores, with the exception of the SEQ, are out of a range of 1–5. The SEQ range is 1–7.

^+^The Net promoter score is also included as 1-item of the 8-item SUPR-Q.

## Discussion

Using a multi-phased process combining participatory human-centered design activities and iterative phases of website development, testing, and adaptation, this study demonstrated that it was feasible and acceptable to create a website to offer relationship-based support to women using or interested in PrEP use for HIV prevention. In addition to high acceptability of the site across both beta and pilot testing phases, women reported finding the content relevant and were willing to share the site more broadly, which may have explained the higher numbers of individuals accessing the site via website analytics.

Overall, we found that the multi-phased process offered key iterative insights regarding website development in terms of technical accessibility, perceived safety, and content understandability from end-users’ perspectives. These led to changes in website design, such as the reconceptualization of the HEART relationship quiz results and changes in images and wording, which the phased approach allowed the team to further test. Although beta testing did not lead to substantially higher acceptability scores, there were important changes to content that could have contributed to maintaining high scores in the pilot stage, where women reviewed the site unobserved and unfacilitated. Additionally, it allowed the study to move forward with the pilot phase with greater confidence. Importantly, working alongside potential end users to co-design and refine the website is also an important practice of research equity that aims to ensure that potential end-users-women-are equal partners in the creation of healthcare products that are acceptable and appropriate to their lifestyles [38,39]. This practice has been noted as well by global frameworks on designing effective digital interventions [40,41]. For that reason, and in combination with both high rates of smartphone/internet access in South Africa, and evidence reviews indicating that digital interventions can effectively address mental and sexual and reproductive health and IPV outcomes contribute to the potential for our intervention to have effect particularly when access to in-person healthcare provision is limited [42–45].

There are some limitations to this work. For one, the website analytic tools and scales used in this study are traditionally used for consumer websites and, as far as we know, have not been used in the African setting nor for the evaluation of more socially / public health-oriented websites. This raises concerns about their validity to assess website user experience in this context. However, they maintain the benefit of being generally comparable across a range of global websites, for example through databases of other website scores [46]. The other concern, related to these measures, is how much social desirability bias plays into the positive results found. This, however, is a concern with all self-reported data and procedures to minimize the ‘white coat effect’ were used. For example, during the pilot research, participants all reviewed the website privately in their own time and for half of these participants, the team conducted surveys over the phone, which may have improved the honesty of survey responses through greater anonymity. Further, the generalizability of our results to broader South Africa are limited by some disparities in smartphone access across the country and the conduct of our study in written English. This is particularly a concern for rural youth, who may have slightly lower access to mobile phone/online access [23,47] and who, in other studies, have identified the lack of native-language content and low digital literacy as barriers to digital mental health interventions [48]. English is, however, commonly used and most media and government activities are conducted in English. This includes government websites targeting young people in the country, such as bwisehealth.org, which CHARISMA aimed to be integrated into. Throughout our development process, we sought and integrated feedback to improve the accessibility of the written English, yet the addition of other languages, as well as more visual rather than written content would continue to improve the website and allow for testing among a larger population of women. Finally, it is worth noting that the expansion of loadshedding–planned power cuts, whose severity are determined by stages, but generally include power cuts for blocks of 2.5 hours–across the country have unequal impacts on individuals in lower socioeconomic strata [49]. This disparity in access to power may limit the reach of digital interventions such as CHARISMA mobile until access to power alternatives, such solar or other alternative sources, become more accessible and affordable.

Although this body of work provided a rigorous adaptation process for translating in-person counselling on PrEP and relationship dynamics into an acceptable mobile website, many features and content suggested by women were unable to be integrated into the final website. Future development and research should continue to integrate more interactive features as desired by women, such as daily mood check-ins, integration of more audio content, the ability to make direct calls/messages and to geolocate service providers, and to share information on social media. Some of these features such as the audio content could make the site more accessible to those with poorer literacy or who lack the patience to read a lot of text. In addition, content catering to males seeking interventions for IPV exposure, to those in same-sex relationships, and to other geographic locations may be needed [50–54]. These adaptations could be accomplished using the existing website structure, which is available as open-source code and as embedded content on the South African Department of Health website, Home—B-Wise (bwisehealth.com) [55]. Finally, future research should evaluate the website’s impact on health and relationship outcomes, such as PrEP uptake and continuation, male partner support for PrEP use, and other key relationship dynamics.

## Conclusion

In settings where IPV and relationship dynamics are key barriers to PrEP use, the CHARISMA mobile site holds promise as a cost-effective, acceptable way to offer relationship support to women using or interested in using PrEP. Developed through a multi-phased participatory process, the current tool is publicly available and should used, adapted, and further tested and refined by health practitioners and researchers, alike, to improve PrEP uptake, adherence, and mitigate related fears and experiences of intimate partner violence.

## Supporting information

S1 File Illustrative design workshop outputs–persona creation and counselling activity adaptation. (DOCX)

S2 File Beta testing data. (XLSX)

S3 File Pilot testing data. (CSV)
