# Supplementary material for: Development of a relationship counselling website to identify and mitigate risk of intimate partner violence in the context of women’s PrEP use
Source: PLOS Digit Health. 2023 Aug 14;2(8):e0000329. doi: 10.1371/journal.pdig.0000329 (PMC10424861; doi:10.1371/journal.pdig.0000329)

**S1. Illustrative design workshop outputs – persona creation and counselling activity adaptation**


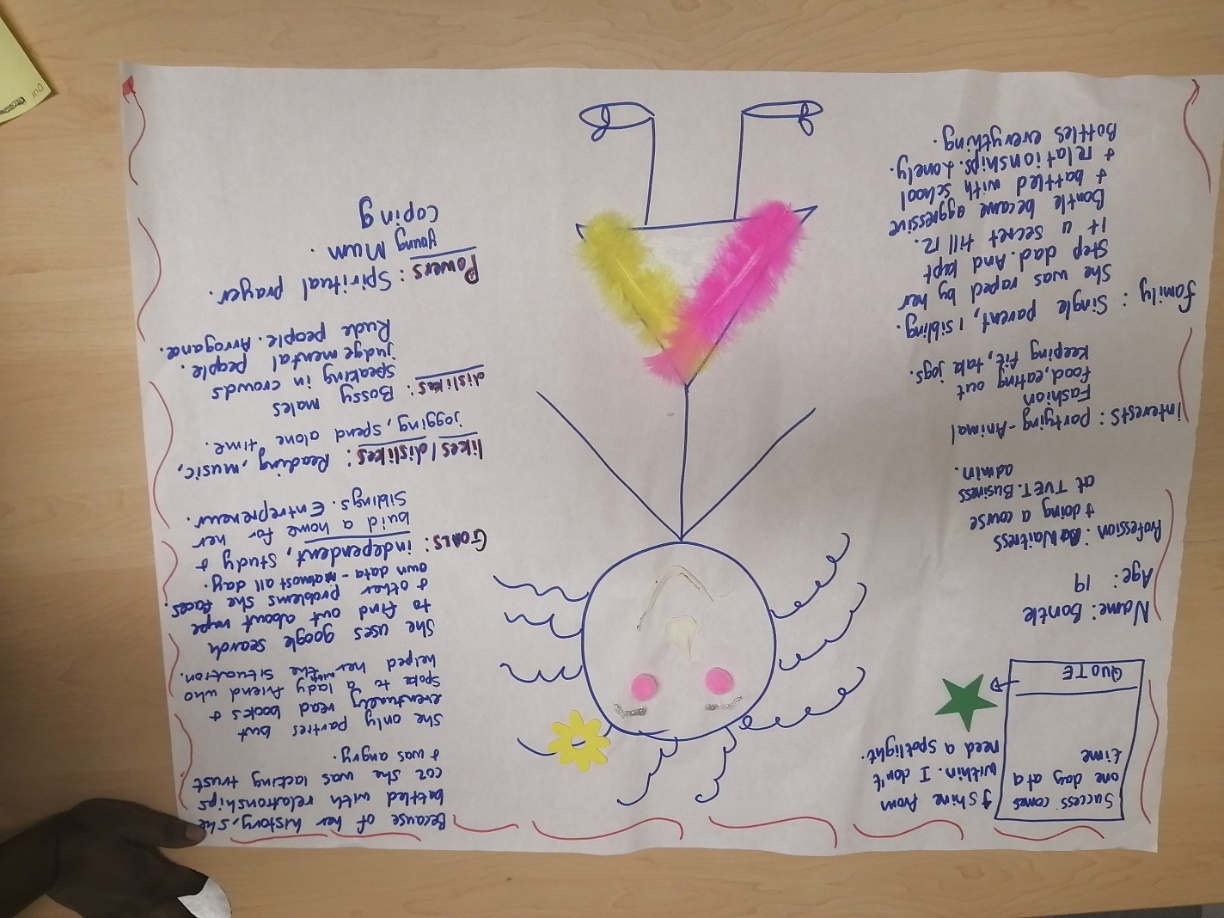


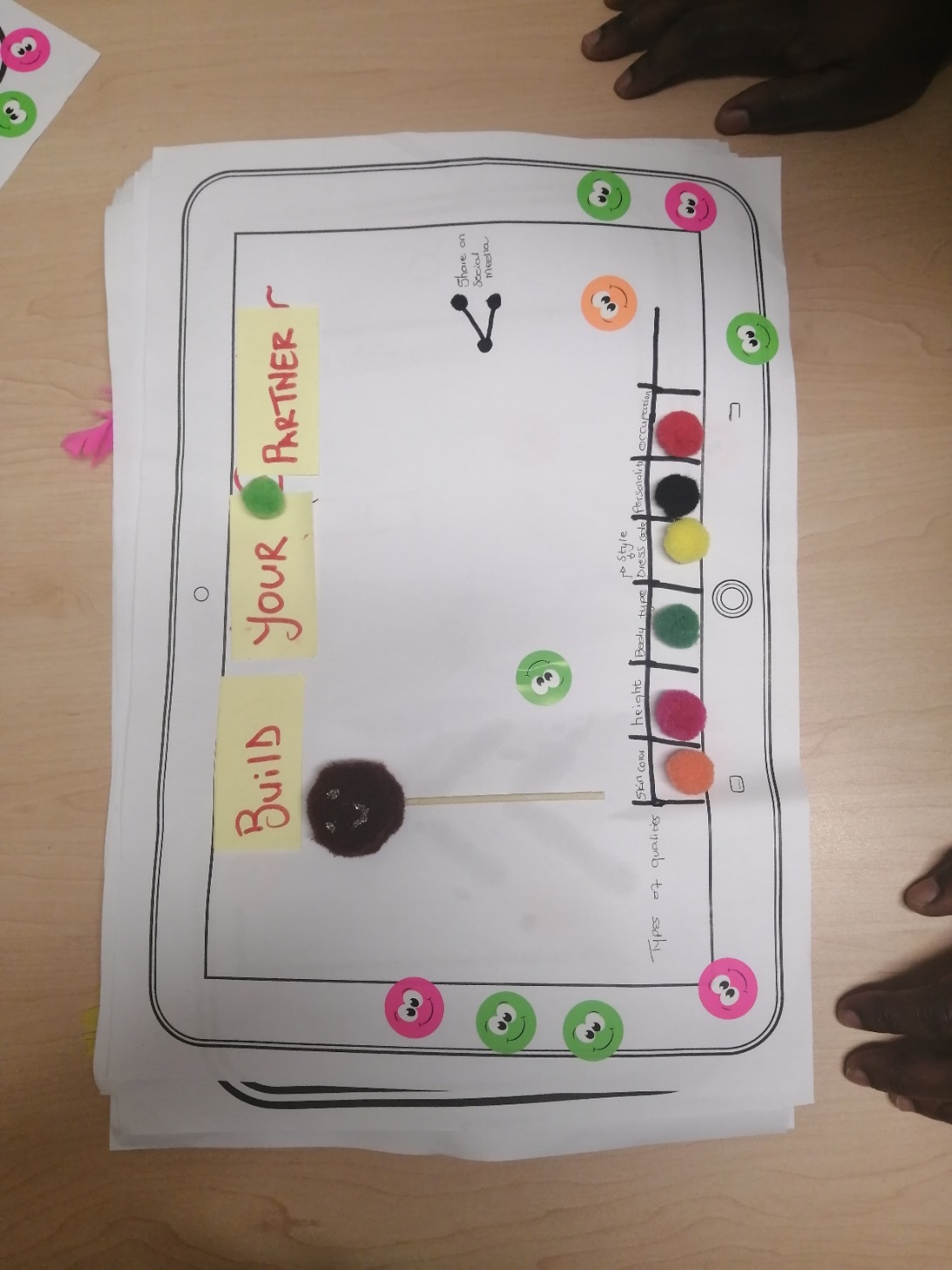


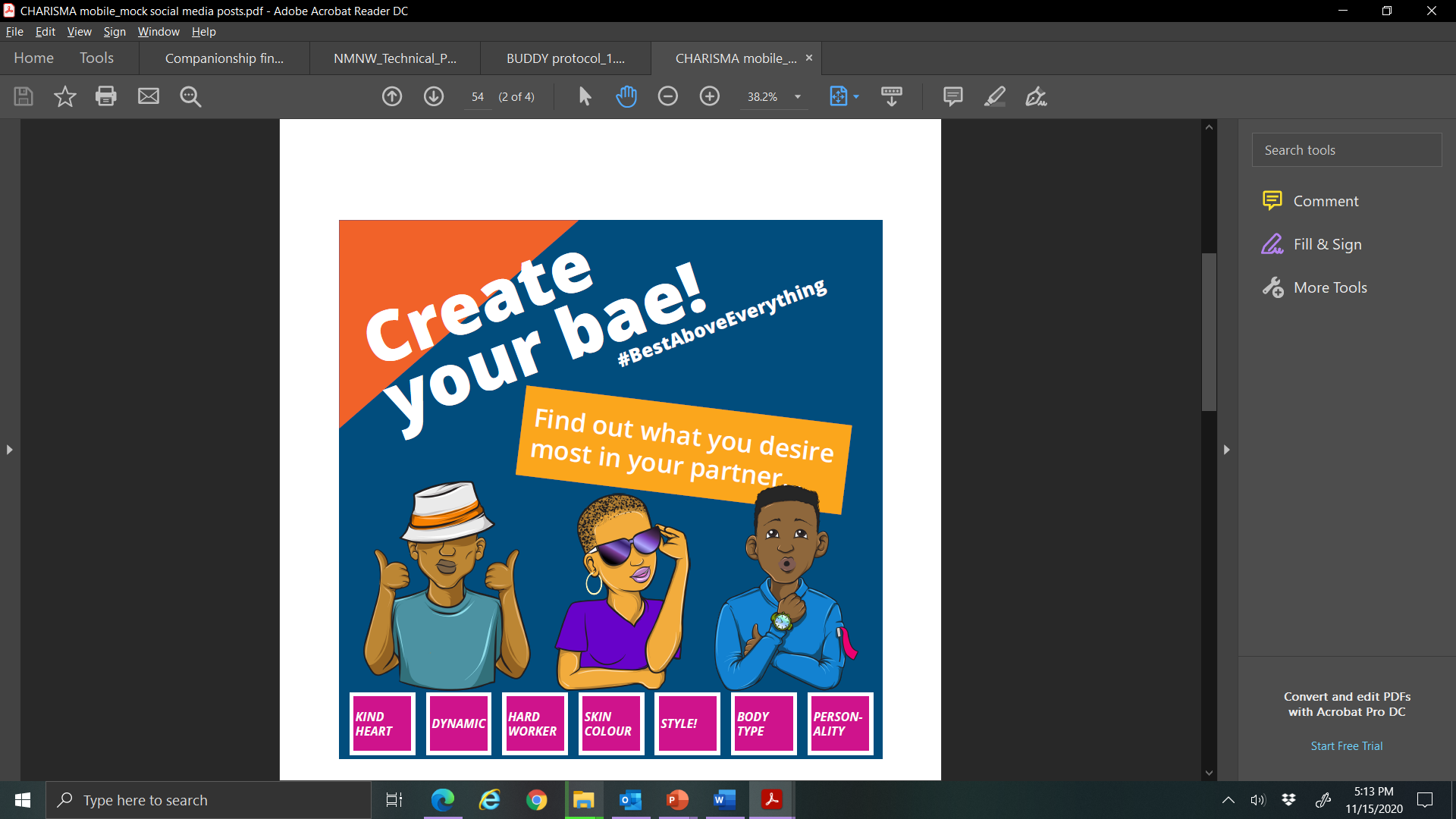


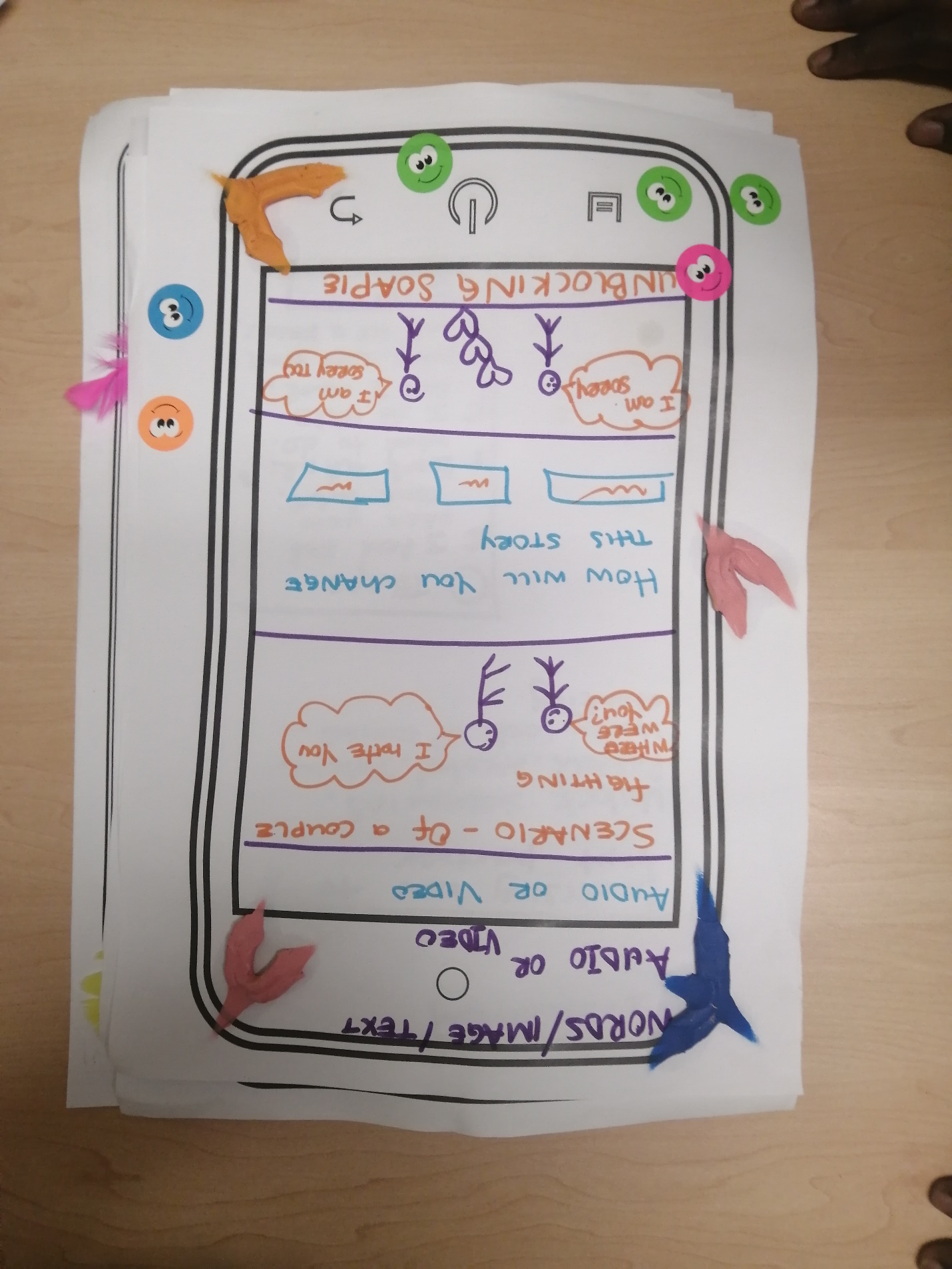

Supplement: S1 File — (DOCX) [file pdig.0000329.s001.docx]
